# Disrupting Dimerization Translocates Soluble Epoxide Hydrolase to Peroxisomes

**DOI:** 10.1371/journal.pone.0152742

**Published:** 2016-05-20

**Authors:** Jonathan W. Nelson, Anjali J. Das, Anthony P. Barnes, Nabil J. Alkayed

**Affiliations:** 1 Department of Anesthesiology & Perioperative Medicine, Oregon Health & Science University, Portland, OR, 97239–3098, United States of America; 2 Department of Molecular and Medical Genetics, Oregon Health & Science University, Portland, OR, 97239–3098, United States of America; 3 The Knight Cardiovascular Institute, Oregon Health & Science University, Portland, OR, 97239–3098, United States of America; 4 Pape Family Research Center, Department of Pediatrics Oregon Health & Science University, Portland, OR, 97239–3098, United States of America; Consejo Superior de Investigaciones Cientificas, SPAIN

## Abstract

The epoxyeicosatrienoic acid (EET) neutralizing enzyme soluble epoxide hydrolase (sEH) is a neuronal enzyme, which has been localized in both the cytosol and peroxisomes. The molecular basis for its dual localization remains unclear as sEH contains a functional peroxisomal targeting sequence (PTS). Recently, a missense polymorphism was identified in human sEH (R287Q) that enhances its peroxisomal localization. This same polymorphism has also been shown to generate weaker sEH homo-dimers. Taken together, these observations suggest that dimerization may mask the sEH PTS and prevent peroxisome translocation. In the current study, we test the hypothesis that dimerization is a key regulator of sEH subcellular localization. Specifically, we altered the dimerization state of sEH by introducing substitutions in amino acids responsible for the dimer-stabilizing salt-bridge. Green Fluorescent Protein (GFP) fusions of each of mutants were co-transfected into mouse primary cultured cortical neurons together with a PTS-linked red fluorescent protein to constitutively label peroxisomes. Labeled neurons were analyzed using confocal microscopy and co-localization of sEH with peroxisomes was quantified using Pearson’s correlation coefficient. We find that dimer-competent sEH constructs preferentially localize to the cytosol, whereas constructs with weakened or disrupted dimerization were preferentially targeted to peroxisomes. We conclude that the sEH dimerization status is a key regulator of its peroxisomal localization.

## Introduction

Soluble epoxide hydrolase (sEH) is considered a therapeutic target for multiple cardiovascular diseases including stroke [1] based on the fact that either gene deletion or pharmacological inhibition of sEH have been shown to be neuroprotective after ischemia [2,3]. Human sEH protein polymorphisms have been associated with either increased risk (K55R) [4] or protection from stroke (R287Q) [5], further strengthening the link between sEH function and stroke risk. Additionally, an *in vitro* model of neuronal ischemia indicated that the R287Q polymorphism conferred increased cell survival [6]. Historically, the effects of both polymorphisms were attributed to how they affect the ability of sEH to hydrolyze the class of neuroprotective fatty acid epoxides called epoxyeicosatrienoic acids (EETs). Indeed, the K55R polymorphism has been shown to increase sEH hydrolase activity, while the R287Q has been shown to decrease its activity [7,8]. However, it has also been shown that the R287Q polymorphism shifts the subcellular localization of sEH from of the cytosol and into the peroxisome [9].

Endogenous sEH exhibits a dual cytosolic and peroxisomal subcellular distribution [10], resulting from a weak C-terminal peroxisome targeting signal (PTS). Interestingly, sEH is the only human protein to end in the Serine-Lysine-Methionine (-SKM) tripeptide sequence, which has lower affinity for the peroxisome transport protein peroxin 5 (Pex5) than the canonical PTS tripeptide Serine-Lysine-Leucine (-SKL) [11]. Indeed, the dual localization of sEH in the cytosol and in peroxisomes can be shifted exclusively to the peroxisomes by changing the sEH C-terminal PTS to -SKL [9]. However, the lower affinity of –SKM does not entirely explain sEH’s dual localization, as recent evidence suggests that the quaternary structure of sEH protein may also regulate the distribution of sEH within the cell [9]. In support of this, Luo et al. demonstrated that enhanced peroxisome localization is observed in Chinese Hamster Ovarian (CHO) cells transfected with sEH protein harboring the single R287Q amino acid substitution [9], previously shown to partially disrupt sEH dimerization [12].

However, the regulation of sEH subcellular distribution in neurons has not previously been investigated. This is an important cell type to understand the mechanism of sEH subcellular distribution given previous studies identifying sEH as a stroke risk factor [3,13]. Furthermore, two mouse studies have suggested that the subcellular distribution of sEH within neurons is linked to the degree of damage in ischemic injury [6,14]. Therefore, we set out to directly test the hypothesis that dimerization regulates the subcellular distribution of sEH in primary neuronal culture. To do this, we leveraged previously validated sEH mutations that alter its dimerization state via perturbation of a dimer-stabilizing salt-bridge [15]. These sEH constructs were fused to GFP to assess their subcellular distribution in primary cortical neurons using laser-scanning confocal microscopy. sEH’s co-localization with peroxisomes was then quantified using Pearson’s correlation coefficient (PCC).

Peroxisome localization of sEH has recently been shown to reduce infarct size in response to stroke [16]. Therefore understanding the factors that contribute to sEH sub-cellular distribution holds the promise of novel therapeutic avenues to reduce neuronal injury following stroke. Specifically, research into the cellular mechanism of sEH targeting offers the possibility of developing strategies that pharmacologically manipulate the subcellular distribution of sEH by mimicking the effect of the R287Q mutation and ultimately improving stroke outcomes.

## Materials and Methods

### Ethics Statement

The investigation conformed to the Association for Assessment and Accreditation of Laboratory Animal Care AAALAC Accreditation (approved July 2013) and the Office of Laboratory Animal Welfare (OLAW Assurance # A3304-01, approved March 2009). The Oregon Health and Science University Animal Care and Use Committee approved all experiments.

### GFP-sEH and RFP-SKL Plasmids

sEH cDNA containing sEH dimerization mutants were amplified using primers that created a 5’-EcoRI and 3’-NotI restriction site for in-frame subcloning into a vector expressing enhanced green fluorescent protein (GFP) driven by elongation factor α (EF1α) promoter [15]. PCR mediated mutagenesis was used to either delete the C-terminal peroxisome targeting signal (GFP-sEH-ΔPTS), or replace it with the canonical PTS1 motif Serine-Lysine-Leucine (GFP-sEH-SKL) (see primers in Table 1). To label neuronal peroxisomes, mCherry fused to the C-terminal canonical PTS1 motif SKL (RFP-SKL) was kindly provided to us by Dr. Tom Maynard.

**Table 1:** GFP-sEH primers. Primer name and sequence used for cloning sEH into GFP expression vector as well as primers used to mutate the C-terminal of sEH to either end in a canonical peroxisome targeting signal (Serine-Lysine-Leucine, SKL) or without a peroxisome targeting signal (ΔPTS). Base pairs in capital letters are restriction endonuclease sequences used for subcloning.

Primer Name	Primer Sequence (5'->3')
EcoRI-sEH-5'	aaaaGAATTCacgctgcgcgcggccgt
sEH-NotI-3'	ttttGCGGCCGCctacatctttgagacca
sEH-SKL-BamHI-NotI-3'	ttttGCGGCCGCGGATCCttacagctttgacttcagcatctttgagaccaccggt
sEH-ΔPTS-SacI-NotI-3'	ttttGCGGCCGCGAGCTCttagaccaccggtgggttccggg

### Primary Cortical Mouse Neuronal Culture

Primary cortical mouse neurons were prepared from C57/BL6 (Charles River) mouse brains at E16, as previously described [17]. Cortices were dissected in HEPES-buffered HBSS and dissociated by digestion with 0.1% papain (Worthington Biochem). Cells were seeded at 600,000 cells/well onto poly-D-lysine (100 μg/ml)-coated 12-well coverslips, and were maintained in a humidified incubator in air with 5% CO_2_. Neurons were cultured in Neurobasal medium without phenol red (Invitrogen), supplemented with 2% B27 (Invitrogen), 1% Glutamax (Invitrogen) and 1% penicillin/streptomycin (Invitrogen). Cultures consisted of >90% microtubule-associated protein 2 (MAP2)-positive and <10% glial fibrillary acidic protein (GFAP)-positive cells on day 10 *in vitro* (DIV 10).

### Plasmid Purification and Transfection

GFP-sEH and RFP-SKL plasmids were purified with a Maxi Kit (Qiagen) according to manufacturer’s protocol and diluted to a final concentration of 900 ng/μL. Lipofectamine 2000 (Invitrogen) was mixed with 900 ng of each plasmid in accordance with the manufactures instructions. All transfections were done on DIV 7 using 1000 μl of supplemented Neurobasal medium for 1 hour, which is then changed to fresh maintenance medium. Cells were fixed 72h after transfection on DIV 10 in 4% paraformaldehyde. Hoechst (Invitrogen) staining of nuclei was performed with 3.2 μM final concentration in PBS for 90 seconds. Slides were then washed and mounted with Fluoromount G (Southern Biotech).

### HEK Cells and Western Blot

Human Embyronic Kidney (HEK) cells were maintained and transfected as previously described [15]. Recombinant sEH and GFP proteins were also synthesized and purified, and Western blot was performed using published protocols [6][15]. Rabbit anti-sEH (Cayman Chemical, 1:400) and rabbit anti-GFP (Cell signaling, 1:1000) antibodies were used as primary antibodies. Cy3-conjugated anti-rabbit antibody (GE Healthcare, 1:2000) was used as a secondary antibody. Immunoblots were detected on a Typhoon Imaging system (GE-Healthcare).

### Image Acquisition

Images were acquired on a Zeiss LSM 510 microscope using a Plan Apochromat 63x/1.4NA oil objective lens unless otherwise noted in figure legend. GFP was excited with a 488-nm laser and detected at 500–530 nm. RFP was excited at a 543-nm laser and detected beyond 560 nm. Laser intensities were optimized for each individual image to take advantage of the full dynamic range of signal. As expected with a transient transfection, each condition contained a mixed population of dim and bright cells. The depth of each neuron was captured with 2048 x 2048 pixel (68 x 68 μm) resolution centered on the nucleus with 16-bit depth coverage. At least 20 images were analyzed for each experimental groups. The specific number of images analyzed for each group is presented in each figure. Images were acquired from at least 3 independently transfected coverslips representing 2 to 5 independent neuronal cultures (Table 2).

**Table 2:** Number of independent cultures, coverslips, and images for each experimental group.

	GFP-sEH Construct						
	WT	ΔPTS	SKL	R287Q	R287E	E254R	R287E + E254R
Number of Cultures	3	4	4	4	5	2	3
Number of Coverslips	3	4	4	4	6	3	3
Number of Images	26	25	24	28	32	21	25

### Image Analysis

Images were analyzed for co-localization with IMARIS (Bitplane) using the co-localization module unless otherwise noted in figure legend. For all GFP-sEH groups, a single representative optical section was used to measure Pearson’s correlation coefficients. Raw images were thresholded equally in the GFP (6.65% signal) and RFP channel (6.80% signal), and then masked in the green channel at (5.69%) before calculating coefficients between the GFP (sEH) and RFP-SKL (demarcating peroxisomes) signals.

### Image Presentation

ZEN software (Zeiss) and Photoshop (Adobe) were used to create images presented in manuscript unless otherwise noted in figure legend. Presented images are maximum intensity projections of a 2 μm sub-stack highlighting a dendritic process and part of the neuronal cell body from the full view images used for analysis. While raw images were analyzed for co-localization, for presentation purposes brightness and contrast have been adjusted in the representative figures.

### Statistics

Data is presented as mean ± sem of Pearson’s correlation coefficient values. To determine if there was a difference in co-localization between wild-type GFP-sEH (GFP-sEH-WT) and different mutant groups, one-way analysis of variance (ANOVA) was performed, followed by Student-Newman-Keuls post-hoc analysis. The number of cultures, coverslips, and images are presented in Table 2.

## Results

### Monitoring sEH subcellular localization through GFP fusion

**Fig 1:**
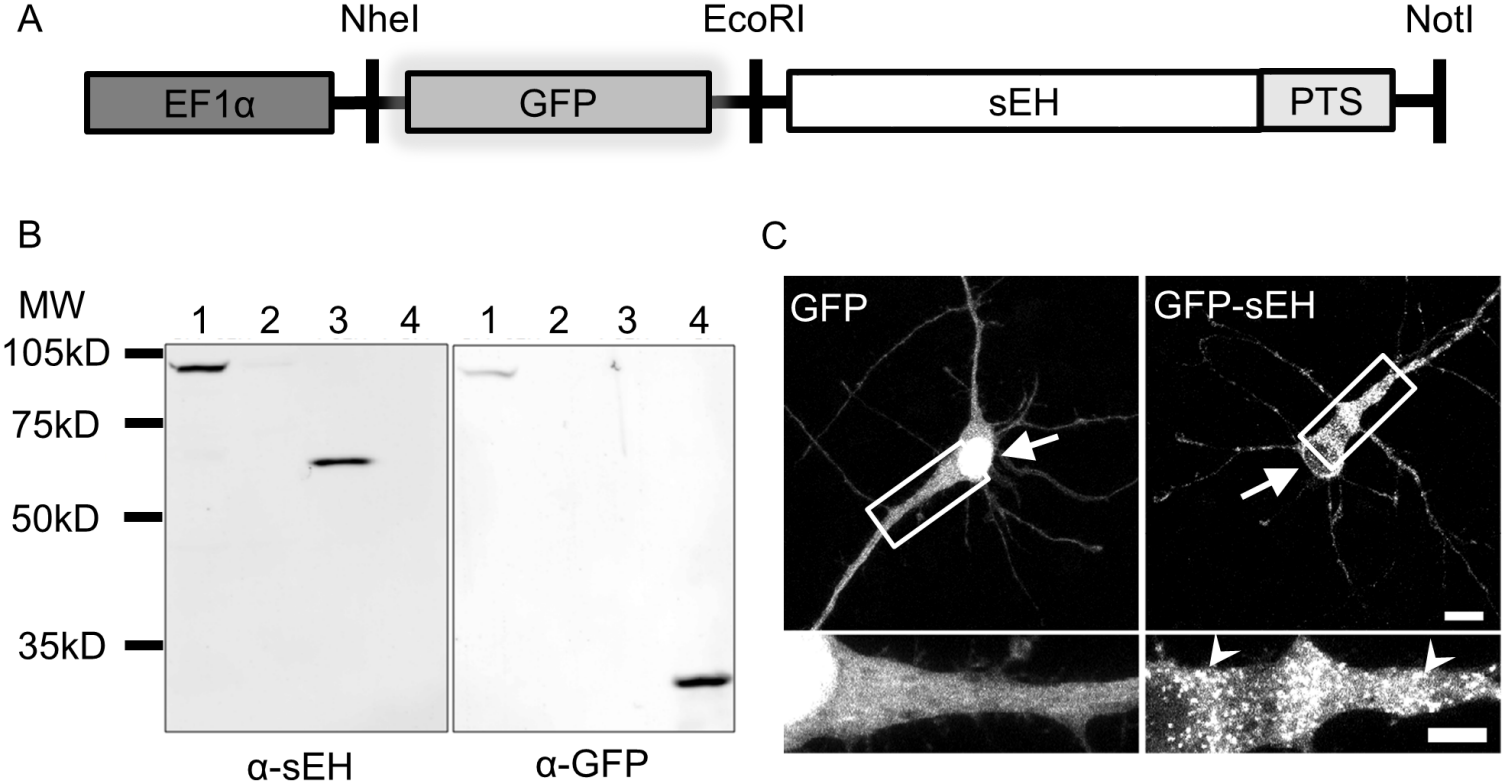
Modeling sEH subcellular localization through GFP fusion. (A) Schematic of GFP-sEH vector. GFP was fused to the N-terminal of sEH in order to preserve the C-terminal peroxisome targeting signal (PTS) of sEH. Expression of GFP-sEH fusion protein is driven by the elongation factor 1-alpha (EF1α) promoter. (B) Western blot of HEK cell lysate transfected with GFP-sEH and recombinant proteins. GFP-sEH has the appropriate molecular weight for a predicted GFP-sEH fusion and is immunoreactive with both anti-sEH (α-sEH) as well as anti-GFP (α-GFP) antibodies. Lane 1: GFP-sEH, Late 2: untransfected HEK cell, Lane 3: recombinant sEH, Lane 4: recombinant GFP. (C) Primary cortical neurons transfected with GFP or GFP-sEH fusion protein. Transfection with GFP results in a diffuse stain throughout the cytoplasm and nucleus of transfected cells. Transfection with GFP-sEH fusion protein, on the other hand, results in both a diffuse and punctate (white arrowheads) pattern. Scalebar on full image is 10 μm and scalebar on insets is 5 μm.

We employed an N-terminal GFP fusion strategy to preserve the C-terminal PTS of sEH that allows us to track the effects of dimerization on the localization of human sEH. This approach parallels a previously study of sEH distribution using heterologous expression of human polymorphisms in Chinese hamster ovarian (CHO) cells [9] (Fig 1A).

Western blot analysis of HEK cell lysates transfected with either GFP-sEH-WT recombinant sEH or GFP proteins was used to confirm expression fusion of GFP-sEH fusions. We found that HEK cells transfected with GFP-sEH-WT produced only a single band that demonstrated immunoreactivity for both anti-sEH and anti-GFP antibodies, similar to the expected size of 90 kD for a GFP-sEH fusion protein (Fig 1B).

We visualized the localization pattern of sEH by transfecting either GFP or GFP-sEH-WT into DIV 7 primary cortical neurons. The cells were allowed to express each protein for 72 hours to reach a dynamic equilibrium of localization before the cells were fixed on DIV 10. In order to obtain high-resolution images, neurons were imaged using laser-scanning confocal microscopy. We found that cells expressing GFP exhibited the expected diffuse staining pattern, whereas neurons transfected with GFP-sEH-WT had a dual staining pattern that is both diffuse and punctate (Fig 1C), consistent with sEH immunoreactivity distribution in other cell types, including human tissue [9,10].

### Quantification of sEH subcellular localization

**Fig 2:**
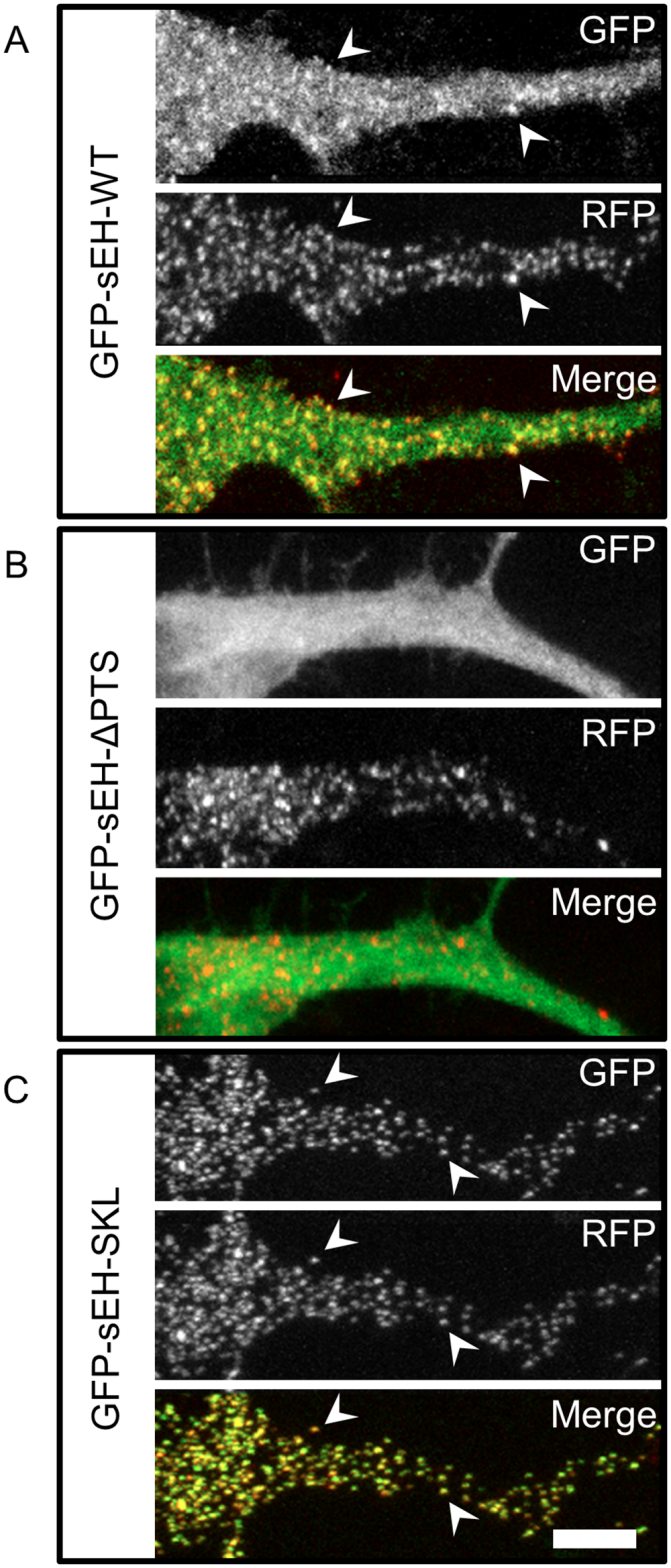
sEH subcellular localization. Primary neurons co-transfected with GFP-sEH fusion protein and peroxisomal marker RFP-SKL. (A) GFP, RFP, and merged image from a neuron transfected with GFP-sEH-WT. GFP-sEH-WT fusion results in a diffuse and punctate stain. GFP-sEH-WT puncta co-localize with RFP-SKL (white arrowheads) reflecting the dual distribution of GFP-sEH-WT between the cytosol and peroxisomes. (B) GFP, RFP, and merged image from a neuron transfected with GFP-sEH-ΔPTS. Deletion of the peroxisomal targeting signal in sEH results in a diffuse localization pattern. (C) GFP, RFP and a merged image from a neuron transfected with GFP-sEH-SKL. Fusion of a canonical peroxisomal targeting signal results in an enhanced punctate pattern that co-localizes with RFP-SKL (white arrowheads), indicative of peroxisomal localization. Scalebar is 5μm.

The addition of C-terminal –SKL peptide to fluorescent proteins has been extensively used to label peroxisomes in living cells [18–20]. Here, we co-transfected RFP fused to the canonical PTS SKL with each GFP-sEH construct to confirm that the punctate appearance of GFP-sEH-WT is the result of sEH translocation into peroxisomes. We found that GFP-sEH-WT partially co-localized with the RFP, indicating peroxisomal localization of a pool of our tagged sEH (Fig 2A, white arrowheads).

Having confirmed that our GFP-sEH-WT construct exhibited dual cytosolic and peroxisomal distribution, we next mutated the PTS of sEH in order to artificially enhance or prevent sEH peroxisomal translocation using PCR mutagenesis. We chose to either prevent sEH from translocating to peroxisomes by deleting the PTS (GFP-sEH-ΔPTS) or enhance sEH peroxisomal translocation by replacing the C-terminus of sEH with the highly efficient canonical PTS (GFP-sEH-SKL).

We found that deletion of the C-terminal PTS of sEH (GFP-sEH-ΔPTS) results in a diffuse pattern similar to neurons transfected with GFP (Fig 2B). In contrast, sEH ending with the C-terminal PTS signal SKL had an exclusively punctate appearance that co-localized with peroxisomes (Fig 2C, white arrowheads) [9]

**Fig 3:**
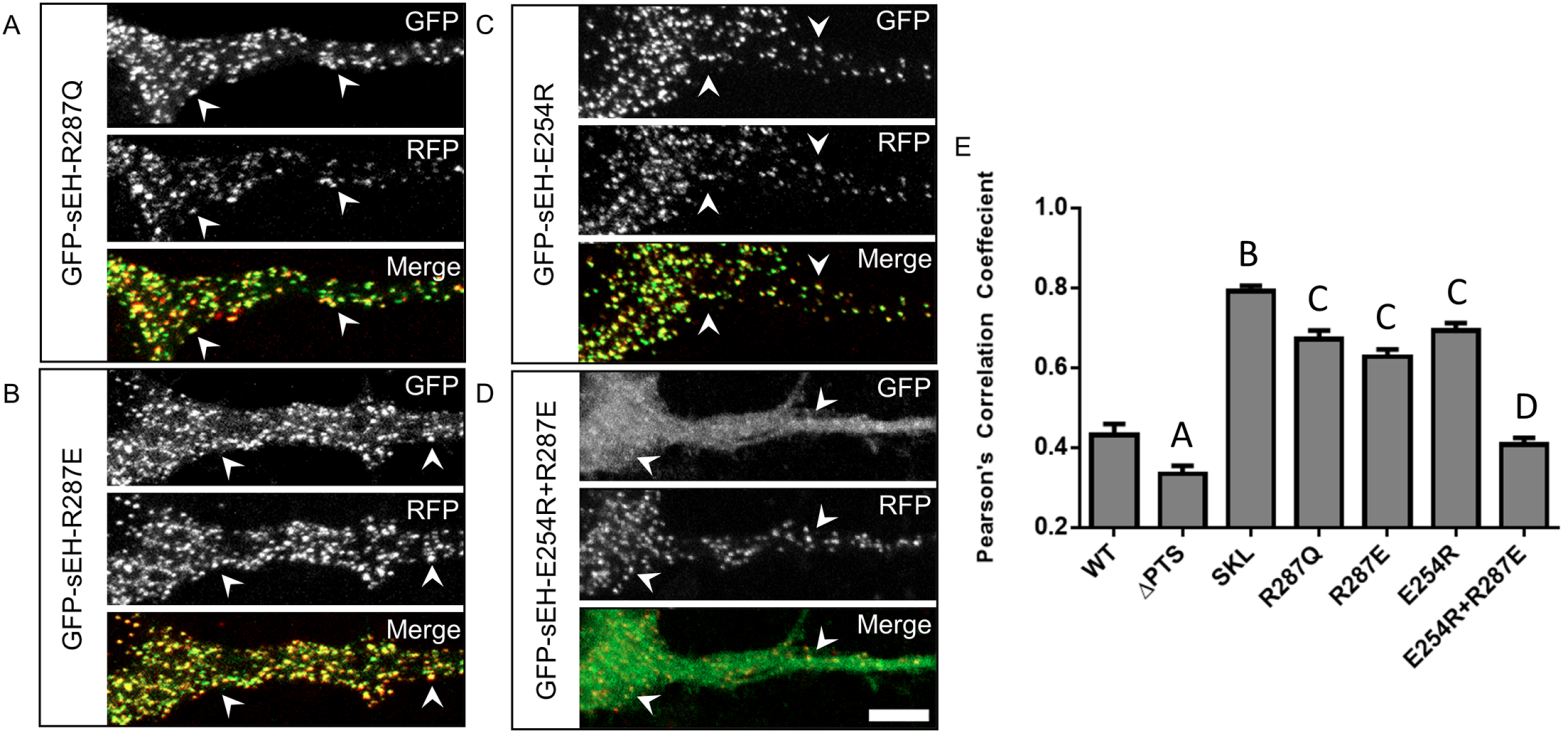
Disrupting dimerization preferentially localizes sEH to peroxisomes while rescuing dimerization restores wildtype sub-cellular distribution. Primary neurons co-transfected with GFP-sEH fusion protein and peroxisome marker RFP-SKL. (A) GFP, RFP and merged image from a neuron transfected with GFP-sEH-R287Q. GFP-sEH-R287Q fusion results in a punctate pattern that highly co-localizes with RFP-SKL (white arrowheads) indicative of peroxisome localization. (B) GFP, RFP and merged image from a neuron transfected with GFP-sEH-R287E. GFP-sEH-R287E fusion results in a punctate pattern that correlates with RFP-SKL (white arrowheads) indicative of peroxisomal localization. (C) GFP, RFP and merged image from a neuron transfected with GFP-sEH-E254R. GFP-sEH-E254R fusion results in a punctate pattern that co-localizes with RFP-SKL (white arrowheads), indicative of peroxisomal localization. (D) GFP, RFP and merged image from a neuron transfected with GFP-sEH-E254R+R287E. GFP-sEH-E254R+R287E fusion results in a dual diffuse and punctate staining pattern. GFP-sEH-E254R+R287E puncta colocalize with RFP-SKL (white arrowheads) reflecting the dual distribution of GFP-sEH-E254R+R287E between the cytosol and peroxisomes similar to GFP-sEH-WT. (E) Quantification of peroxisome localization for each GFP-sEH construct with Pearson’s correlation coefficient. A indicates p<.05 compared with WT, SKL, R287E, and E254R. B indicates p<.05 compared with WT, ΔPTS, R287Q, R287E, and E254R and E254R+R287E. C indicates p<.05 compared with WT, ΔPTS, and E254R+R287E. D indicates p<.05 compared with ΔPTS, SKL, R287Q, R287E, and E254R. Scalebar is 5μm.

These distinct localization patterns allowed us to develop and validate a quantification system for the distribution of sEH between the cytosol and peroxisomes using Pearson’s correlation coefficient (PCC) by comparing the distribution GFP-sEH-WT and the peroxisome labeling protein RFP-SKL. PCC is an established measure of co-localization between two signals that ranges from -1 (perfect exclusion) to 1 (perfect co-localization) [21]. Importantly, PCC measures the variability between two signals and not the absolute intensity of signals. Therefore, PCC should not be sensitive to variability in expression level due to variability in transfection efficiency. We found that GFP-sEH-WT had a PCC of 0.43±.03. Deletion of the C-terminal PTS of sEH (GFP-sEH-ΔPTS) yielded a significant decrease in PCC to 0.33±.02 (p<.05 compared to GFP-sEH-WT), conversely changing the PTS to SKL significantly increases PCC to 0.79±.01 (p<.05 compared to GFP-sEH-WT) (Data shown in Fig 3E). While the GFP-sEH-ΔPTS expression pattern appeared diffuse with no noticeable co-localization with peroxisomes, images were quantified to have a mean PCC value of .33. This value may be slightly elevated above 0 because of a basal level of signal overlap within the cells that are co-transfected with both RFP and GFP.

### Disrupting dimerization preferentially localizes sEH to peroxisomes

Our GFP-sEH transfection model coupled with PCC quantification of sEH peroxisomal localization allowed us to test the hypothesis that dimerization regulates the distribution of sEH between the cytosol and peroxisomes. Previously, a human polymorphism in sEH (R287Q) was shown to partially disrupt the homodimerization of sEH [12]. This specific polymorphism has also been shown to preferentially localize to the peroxisome compared to the WT protein in CHO cells, leading the authors of that study to hypothesize that dimerization was regulator of the subcellular distribution of sEH [9]. Consistent with this previous work, we find that sEH harboring the R287Q polymorphism (GFP-sEH-R287Q) forms a punctate pattern that more strongly co-localizes with peroxisomes in neurons (Fig 3A).

We previously identified and characterized additional systematic mutations of amino acids forming an essential dimer-stabilizing salt-bridge (E254 and R287) that control the dimerization state of sEH [15]. We further demonstrated that these mutations either completely disrupt or rescue sEH dimerization. Specifically, we demonstrated that both E254R and R287E mutations severely disrupt sEH dimerization, whereas combining the two mutations (E254R+R287E) rescues dimerization by inverting the dimer-stabilizing salt-bridge [15]. Here, we took advantage of these highly characterized sEH constructs to further test the hypothesis that dimerization regulates the subcellular distribution of sEH. We found that transfection with either GFP-sEH-R287E (Fig 3B) or GFP-sEH-E254R (Fig 3C) results in a punctate signal that co-localizes with peroxisomes (Fig 3B and 3C, white arrowheads).

We found that dimer-disrupting mutations significantly increase peroxisome localization as measured by PCC from 0.43±.03 (GFP-sEH-WT) to 0.67±.02, 0.63±.02, and 0.69±.02 for GFP-sEH-R287Q, GFP-sEH-R287E and GFP-sEH-E254R respectively (Fig 3E). Interestingly, while the peroxisome localization of disrupted dimerization constructs did not differ greatly from each other, they all were significantly less than GFP-sEH-SKL (p<.05).

In contrast to disrupting dimerization, rescuing sEH dimerization, by combining the E254R and R287E mutations (GFP-sEH-E254R+R287E), results in a dual diffuse and punctate pattern in which the punctate signal co-localizes with peroxisomes (Fig 3D, white arrows). Quantification of neurons transfected with GFP-sEH-E254R+R287E reveals that it is statistically different from cytosolic sEH (p<.05 compared to GFP-sEH-ΔPTS) and dimer-disrupted sEH (p<.05 compared to GFP-sEH-R287Q, GFP-sEH-R287E, and GFP-sEH-E254R+R287E), but statistically indistinguishable from GFP-sEH-WT (0.43±.03 vs. 0.41±.02). (Fig 3E).

### Effect of sEH expression on subcellular localization in neurons

**Fig 4:**
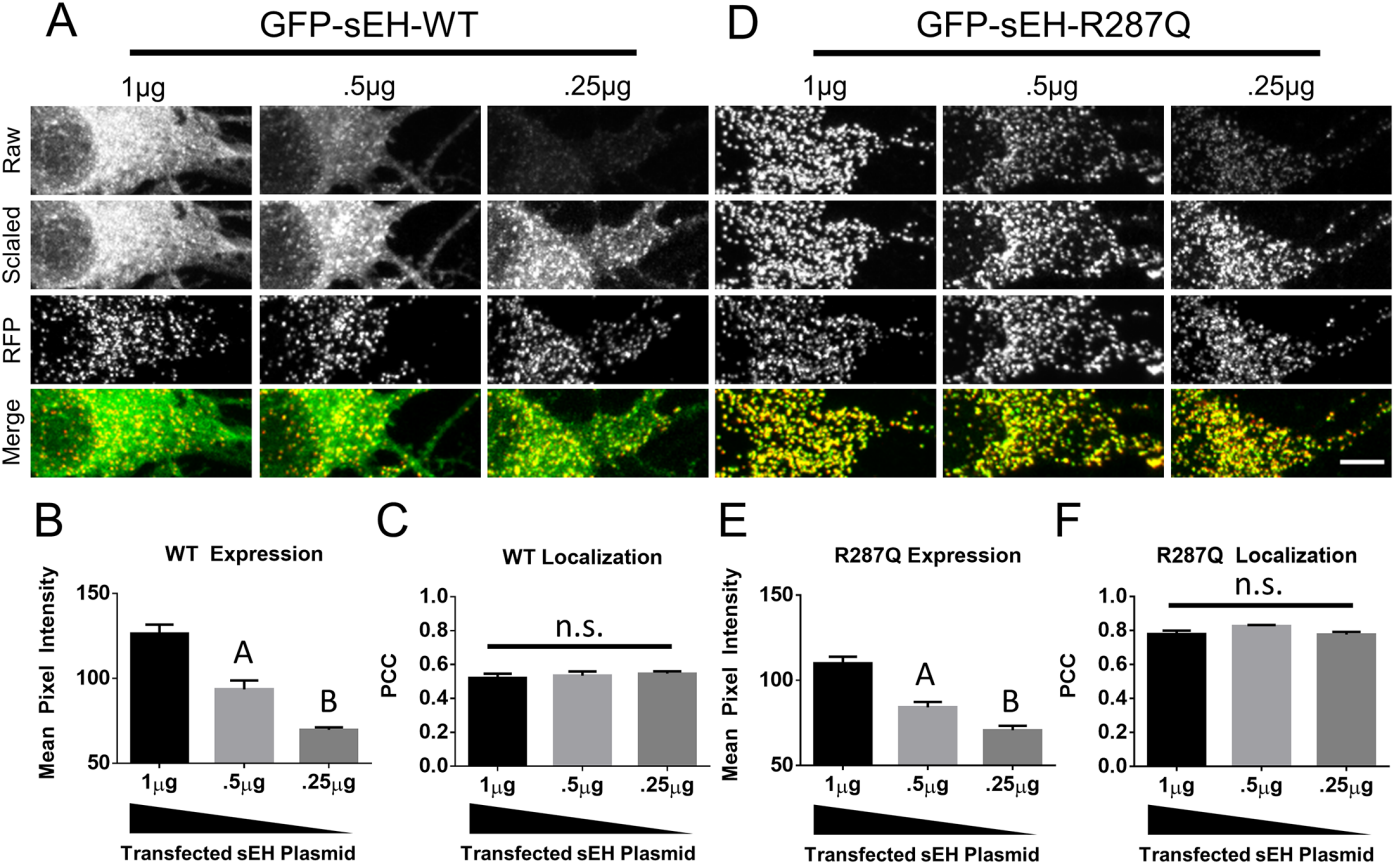
Effect of sEH expression on subcellular localization. (A) Primary neurons co-transfected with different amounts of GFP-sEH-WT plasmid. Decreasing amounts of transfected plasmid results in reduced expression of GFP-sEH-WT as can be seen in the raw images and in (B) measurements of mean pixel intensity. (C) Measurement of colocalization between GFP and RFP signals. D. Primary neurons co-transfected with different amounts of GFP-sEH-R287Q plasmid. Decreasing amounts of transfected plasmid results in reduced expression of GFP-sEH-R287Q as can be seen in the raw images and in (E) measurements of mean pixel intensity. (F) Measurement of colocalization between GFP and RFP signals. Raw images were collected under the same image capture settings, scaled images are the contrasted images used to create the Merge image with the RFP image which labels peroxisomes. Images from this figure were acquired on a Nikon A1R Laser Scanning Confocal Microscope and analyzed for mean pixel intensity and Pearson’s correlation coefficient using Elements (Nikon). At least 10 different images from 2 independent cultures were used for each condition. A indicates p<.05 compared with 1μg and .25μg. B indicates p<.05 compared with 1μg and .5μg. n.s. indicates not significant. Scalebar is 5μm.

A previous study presented evidence that in addition to dimerization, expression level of sEH may also be a contributing factor in its subcellular distribution [9]. To determine whether expression level of sEH influences the subcellular distribution of sEH in mouse primary cortical neurons we transfected neurons with differing amounts of GFP-sEH plasmid and constant amount of RFP targeted to peroxisomes and co-localized the GFP signal with a the RFP signal. In contrast to the previous study, we find that despite decreasing the expression level of GFP-sEH WT (Fig 4A and 4B) the co-localization of GFP-sEH-WT with RFP targeted to peroxisomes remains constant (Fig 4C). However, similar to the previous study, we found that decreasing the expression of GFP-sEH-R287Q does not change the co-localization with RFP targeted to peroxisomes.

## Discussion

Soluble epoxide hydrolase is a promising therapeutic target for stroke, with interesting biology, protein structure and subcellular localization. Previous work with a human polymorphism of sEH (R287Q) suggested a relationship between dimerization of sEH and its subcellular localization [9]. Here we extend those findings by systematically testing the hypothesis that dimerization regulates the subcellular distribution of sEH between the cytosol and peroxisomes in neurons using previously validated sEH mutants that either disrupt or rescue dimerization [15].

Here, we find that two different constructs of sEH previously shown to form dimers (GFP-sEH-WT and GFP-sEH-R287E+E254R) co-localize with a peroxisome marker less than three sEH constructs (GFP-sEH-R287Q, GFP-sEH-R287E, GFP-sEH-E254R), which disrupt dimerization. We believe this to be a compelling evidence that dimerization plays a central role in the regulation of sEH subcellular localization. Interestingly, there was no increase in peroxisomal localization of GFP-sEH-R287E and GFP-sEH-E254R compared to GFP-sEH-R287Q, despite the fact that R287E and E254R mutations were shown to disrupt dimerization more significantly than the R287Q polymorphism. While, the R287Q polymorphism was measured to have a ratio of 60% dimer and 40% monomer, almost no dimer formation was detected for E254R and R287E mutations [12,15]. This would suggest that only a slight disruption in sEH dimerization is necessary to significantly shift the localization to favor peroxisomes and that there is a ceiling to how well the –SKM PTS can translocate proteins to peroxisomes.

A previous study showed that in addition to dimerization, expression level may contribute to the subcellular distribution of sEH within cells. This conclusion was based on the observation that a stable cell line of CHO cells expressing low levels of GFP-sEH-WT did not have evidence of puncta indicative of peroxisome localization paired with the observation that CHO cells sorted by flow cytometry for the low expression of GFP-sEH-WT also did not have any evidence of puncta indicative of peroxisome localization. To test whether expression of sEH contributes to the subcellular distribution of sEH within primary cortical mouse neurons we transfected neurons with different amounts GFP-sEH and co-localized GFP-sEH to a RFP peroxisome marker. Our results suggest that expression level does not affect the subcellular distribution of GFP-sEH-WT or GFP-sEH-R287Q. The discrepancy in our results compared to the previous publication may simply reflect a biological difference between CHO cells and mouse primary cortical neurons. Alternatively, the previous paper was able to observe cells with a 10-fold difference in expression which is a larger dynamic range then we were able to obtain in our study. This study has a number of limitations. First, this study extrapolates the dimerization status of each sEH construct based on previous *in vitro* findings [15]. Given the difficulties expressing proteins in primary cultured cortical neurons it is not feasible to measure the dimerization status of the constructs specifically in neurons. Additionally, this study assumes that eGFP is having a negligible effect on sEH dimerization. Based on the domain-swapped architecture of sEH and the orientation of its N-terminus, it is unlikely that eGFP is inhibiting dimerization. Additionally, if eGFP is enhancing dimerization, it would be enhancing dimerization for all sEH construct uniformly. Moreover, an N-terminal eGFP tag has previously been used to study sEH subcellular localization [9].

Our findings in primary neurons strengthen the model of sEH peroxisomal translocation which proposes that in its dimerized form the endogenous PTS of sEH and the peroxisome transport protein peroxin 5 (Pex5) have unfavorable binding conditions resulting in a majority of sEH to be localized to the cytosol. However, as a monomer, the binding conditions of SKM and Pex5 become more favorable resulting in more efficient translocation into peroxisome [9].

Generally, protein localization is a key determinant of function, and protein mis-localization is associated with multiple diseases [22]. The predominantly recognized function of sEH is the metabolism and inactivation of the lipid signaling molecule epoxyeicosatrienoic acids (EETs). EETs are either formed *de novo* by microsomal cytochrome P450 epoxygenases or released from the plasma membrane into the cytosol. In both cases, they are further metabolized into their diol metabolites in the cytosol by the action of sEH.

On the other hand, the role sEH plays in peroxisomes remains unclear. It is known that peroxisomes play a key role in the metabolism of reactive oxygen species (ROS) within the cell [23]. Under stressful conditions leading to peroxisome dysfunction, such as ischemia/reperfusion injury, a defect in ROS metabolism may lead to increase in the formation of toxic fatty acid epoxides within peroxisomes. This has led to the speculation that the peroxisomal epoxide hydrolase activity acts to detoxify these products [24]. This role for sEH would be consistent with the protection from ischemia afforded by R287Q human polymorphism [5,6]. Indeed, the a recent publication has suggested that soluble epoxide hydrolase localized to peroxisomes is protective in ischemia [16]. This action may be protective through two independent mechanisms; 1) sequestration of the enzyme away from EETs in the cytosol thereby elevating levels of this protective molecule and 2) detoxification of fatty acid epoxides in dysfunctional peroxisomes.

In conclusion, this work described here suggests that disrupting sEH dimerization results in enhanced translocation of the enzyme to peroxisomes. This places new emphasis on the role that sEH plays in peroxisomes especially in the context of the clinical phenotypes associated with R287Q human polymorphism [5,25–27]. Our study provides the groundwork for additional inquiries into a mechanism to disrupt sEH dimerization in the general patient population to drive peroxisomal localization and improve outcomes of stroke and ischemia.
